# Prediction of Vigilant Attention and Cognitive Performance Using Self-Reported Alertness, Circadian Phase, Hours since Awakening, and Accumulated Sleep Loss

**DOI:** 10.1371/journal.pone.0151770

**Published:** 2016-03-28

**Authors:** Eduardo B. Bermudez, Elizabeth B. Klerman, Charles A. Czeisler, Daniel A. Cohen, James K. Wyatt, Andrew J. K. Phillips

**Affiliations:** 1 Morehouse School of Medicine, Atlanta, Georgia, United States of America; 2 Division of Sleep and Circadian Disorders, Brigham and Women's Hospital and Harvard Medical School, Boston, Massachusetts, United States of America; 3 Sentara Healthcare, Norfolk, Virginia, United States of America; 4 Rush University Medical Center, Chicago, Illinois, United States of America; University of Oxford, UNITED KINGDOM

## Abstract

Sleep restriction causes impaired cognitive performance that can result in adverse consequences in many occupational settings. Individuals may rely on self-perceived alertness to decide if they are able to adequately perform a task. It is therefore important to determine the relationship between an individual’s self-assessed alertness and their objective performance, and how this relationship depends on circadian phase, hours since awakening, and cumulative lost hours of sleep. Healthy young adults (aged 18–34) completed an inpatient schedule that included forced desynchrony of sleep/wake and circadian rhythms with twelve 42.85-hour “days” and either a 1:2 (n = 8) or 1:3.3 (n = 9) ratio of sleep-opportunity:enforced-wakefulness. We investigated whether subjective alertness (visual analog scale), circadian phase (melatonin), hours since awakening, and cumulative sleep loss could predict objective performance on the Psychomotor Vigilance Task (PVT), an Addition/Calculation Test (ADD) and the Digit Symbol Substitution Test (DSST). Mathematical models that allowed nonlinear interactions between explanatory variables were evaluated using the Akaike Information Criterion (AIC). Subjective alertness was the single best predictor of PVT, ADD, and DSST performance. Subjective alertness alone, however, was not an accurate predictor of PVT performance. The best AIC scores for PVT and DSST were achieved when all explanatory variables were included in the model. The best AIC score for ADD was achieved with circadian phase and subjective alertness variables. We conclude that subjective alertness alone is a weak predictor of objective vigilant or cognitive performance. Predictions can, however, be improved by knowing an individual’s circadian phase, current wake duration, and cumulative sleep loss.

## Introduction

Sleep loss causes deterioration in cognitive performance, an increase in errors, and diminished job performance in many occupations such as medical residents [1,2], pilots [3], commercial drivers [4], and shift-workers [5,6]. A single night of reduced sleep causes lapses in vigilant attention and is a significant causative factor in traffic accidents [7,8]. Chronic sleep restriction (i.e., multiple nights with insufficient sleep), leads to cumulative deterioration in vigilant attention and cognitive performance [9]. To improve public safety, it is important to identify when individuals are at risk for attentional lapses and impaired performance related to sleep loss. As an initial step to devise tools to flag such individuals, we quantified how different metrics predicted vigilant attention and cognitive performance.

Prior investigations have demonstrated changes in vigilant attention and cognitive performance with sleep restriction and circadian phase [10–13]. A meta-analysis of 19 research studies showed impairment in cognition, motor skills, and mood with sleep restriction [14]. Sleep restriction can cause global decreases in brain activity that adversely affect attention [15,16]. An increased duration of wakefulness is associated with greater adverse effects on vigilant attention [9,17,18] and with increasing impairment in psychomotor vigilance as chronic sleep loss accumulates [9,10,14,18,19]. Circadian phase modulates vigilant performance, with improvements during the circadian day and declines during the circadian night [12,19].

While many individuals rely on their self-perceived level of alertness to determine if they can safely perform a task, group average data show a mismatch between objective performance and subjective ratings of alertness [9,18,20]. Since sleep restriction impairs cognition, we hypothesized that it may also affect the ability of an individual to accurately assess his or her level of performance. In studies in which subjective alertness and objective performance were both assessed, the time courses for the group averages of these metrics were different, suggesting that research volunteers were only partially cognizant of the effect of sleep restriction on performance [19,21–23]. Subjective ratings of alertness plateau within a few days of chronic sleep restriction, whereas cognitive performance continues to decline across weeks [9,18]. Furthermore, sleep restricted participants tend to overestimate their cognitive performance during biological night hours [24]. However, none of these studies examined the relationship between subjective alertness and cognitive performance within each testing session for each individual. In other words, it remains unknown how well an individual’s subjective sleepiness predicts attentional failure or other cognitive decline at that moment.

In this study, we investigated whether a similar discrepancy between subjective alertness and vigilant performance is found under forced desynchrony conditions with and without chronic sleep restriction. Then, we quantified within each testing session the extent to which four experimentally-proven important variables—self-perceived rating of subjective alertness (*Alertness*), circadian phase (*Phase*), hours since awakening (*Wake Duration*), and hours of accumulated sleep loss (*Sleep Debt*)–predicted vigilant attention as measured by the Psychomotor Vigilance Task (*PVT*), Addition/Calculation Test (ADD), and the Digit Symbol Substitution Test (DSST). Models that included all possible combinations of each of these four candidate explanatory variables were investigated to find the most predictive variables.

## Materials and Methods

### Ethics

This study was approved by the Partners Healthcare Institutional Review Board. Written informed consent was obtained from all participants after all elements of the experiment protocol were explained in person by an investigator according to the standards of the Human Research Committee of the Brigham and Women’s Hospital, in accordance with the Declaration of Helsinki.

### Participants and screening procedures

Data for these analyses were from 17 participants (4F, 13M; 18 to 34 years) obtained from two inpatient forced desynchrony (FD) protocols: a Control protocol [25] and a Chronic Sleep Restriction (CSR) protocol [26]. Details of these protocols are provided in the referenced publications and summarized below.

1) Common to both protocols: All participants were free of medical illness, sleep disorders, or psychopathology, as verified by detailed medical history, complete physical examination, standard urine and blood screening studies, and psychological evaluation. Participants had no history of shift work, or transmeridian travel for the preceding 3 months Participants were instructed to avoid all over-the-counter medications, alcohol, caffeine, or illicit substances for three weeks before the study. This was verified by urine and blood testing during screening and on admission to the study. Participants were excluded if self-reported habitual sleep durations fell outside of 6.5 to 9 hours. Participants were scheduled to a regular sleep-wake schedule at the same (habitual) time each night for three weeks prior to admission. Participants were allowed to deviate by more than 30 minutes from their habitual sleep times on a maximum of two occasions. These times were verified by sleep logs, time-stamped voice messages, and wrist actigraphy. Participants were studied within the Intensive Physiological Monitoring Unit of Brigham and Women’s Hospital within private suites free of any time cues. All events were scheduled relative to each individual’s habitual bedtimes.

Testing included: (i) An objective metric, the 10-minute Psychomotor Vigilance Task (PVT), where participants are repeatedly presented with a simple visual stimulus and asked to respond as quickly as possible [27]. The PVT is a task requiring both sustained attention and speed, which is a fundamental component of complex tasks [28,29]. Participants’ responses in milliseconds were recorded. Individuals receive feedback on each PVT in that the stimulus is a visual presentation of the number of msec since stimulus onset. Therefore each individual knows their response time for each stimulus presentation. (ii) A second objective metric, the Addition/Calculation Test (ADD), measures cognitive throughput. Responses were recorded as the number of correct answers given in 2 minutes on a 2-digit addition test [25]. Individuals receive a new ADD test each time and do not receive feedback on their responses. (iii) A third objective metric, the 2 minute Digit Symbol Substitution Test (DSST), where a participant uses a key to enter the symbol which corresponds to a presented digit [25]. The participant has to pair the number with the symbol as rapidly as possible, testing vigilant attention, processing speed, memory, and motor skills. Responses were recorded as number of correct answers. Individuals receive a new DSST each time and do not receive feedback on their responses. (iv) A subjective metric, the Visual Analog Scale (VAS), was used to rate *Alertness*. Participants rated their perceived level of subjective alertness on a non-numeric scale, (with “Sleepy” and “Alert” as anchor texts) that was later scored from 0 to 100, where 100 is the highest level of subjective alertness [30].

For the FD portion of the protocol, participants were scheduled to 42.85 hours “days”. A 42.85 hour “day” was used (i) because it is outside the range of entrainment of the circadian pacemaker, whose intrinsic rate is approximately 24.2 hours [31], allowing study of the effects of circadian phase independently of length of time awake, and (ii) so extended wake episodes could be investigated.

2) Control protocol: Eight male volunteers (ages 18–30 years) were scheduled to a FD protocol with a 1:2 sleep:wake ratio. The scheduled sleep opportunity for the 3 weeks prior to entry to the inpatient portion of the protocol was 8 hours. In the inpatient portion of the protocol, each participant was first scheduled to maintain their outpatient schedule for three days. Next, participants began the FD protocol in which each “day” included 28.57 hours of wakefulness and 14.28 hours of sleep opportunity to achieve a sleep:wake ratio of 1:2. This protocol continued for 14 “days”. Light levels were less than 15 lux during wake hours and less than 0.03 lux during scheduled sleep hours to minimize resetting of the circadian rhythm [32]. During the scheduled wake episodes, VAS was collected every 30 minutes and a 25-minute test battery that included PVT, ADD and the DSST occurred every 2 hours.

3) Chronic Sleep Restriction (CSR) protocol: Nine volunteers, (4F, 5M; ages 21–34 years), were scheduled to a FD protocol with a 1:3.3 sleep-wake ratio. The scheduled sleep opportunity for the 3 weeks prior to entry to the inpatient portion of the protocol was 10 hours. In the inpatient portion of the protocol, each participant was first scheduled to three days with 12 hours sleep opportunity during the night and 4 hours sleep opportunity during the day to minimize any prior sleep debt. Participants' next schedule included 10 hours sleep opportunity for 2 days. Then, participants began the FD protocol, in which each “day” included 32.85 hours of wakefulness and 10 hours of sleep opportunity to achieve a sleep:wake ratio of 1:3.3, equivalent to 5.6 hours sleep per 24. This protocol continued for 12 “days”. Technicians were in the room during scheduled wake episodes to minimize inadvertent sleep during scheduled wake hours. Light levels were approximately 4 lux during wake episodes and 0 lux during scheduled sleep times to minimize resetting of the circadian rhythm. During the scheduled wake episodes, VAS was collected every 30 minutes. Two hours after awakening, participants began the first 25-minute test battery which included the PVT every 4 hours, and the ADD and DSST every 2 hours during scheduled wake episodes.

### Primary analyses

Circadian phase (0 to 360 degrees) and period for each participant were calculated using Non-Orthogonal Spectral Analysis (NOSA) of plasma melatonin data, which were collected every 60 minutes throughout the FD protocol [26]. Data from the first 12 FD “days” of the Control protocol (3 weeks) and all 12 FD “days” of the CSR were used. Zero degrees was defined as the fit melatonin maximum (corresponding to ~ 3 am for an individual sleeping habitually between the hours of 11 pm to 7 am). Due to the slightly different conditions prior to FD in each experiment, we analyzed the data from each experiment separately.

### Secondary analyses

Objective (PVT, ADD, and DSST) and subjective (VAS) measurements were paired for these analyses if the tests occurred within 45 minutes of each other. If two VAS tests occurred within 45 minutes of the objective test, the test pair with the shortest time separation was used. Data were binned according to the NOSA-calculated circadian phase of the paired tests. For the purposes of analysis, we defined “Adverse” and “Optimal” circadian phases for performance, using the same definitions as in Cohen et al. [26]. Adverse Phase occurred during the biological night and was defined as -30 to 90 degrees, while Optimal Phase occurred during the biological day and was defined as 150–270 degrees. We also defined “Adverse” and “Optimal” durations of wakefulness for performance: Adverse Time was defined as 20–28 hours after scheduled wake, while Optimal Time was defined as 2–10 hours after scheduled wake.

Data were binned according to week of FD, with one week corresponding to four 42.85-hour “days” (171.4 hrs), which is very close to the duration of a calendar week (168 hours). (ii) Within each week, the four scheduled wake episodes each began at a different circadian phase, resulting in four different combinations of circadian phase and length of time awake for each testing session. This allowed us to investigate the independent contributions of these factors to performance and alertness.

Control and CSR data were also analyzed as a function of the cumulative lost hours of sleep at the time of each test on each protocol. This was calculated as the cumulative number of hours of scheduled wakefulness during the FD protocol up to the time of each test, minus the number of hours the participant would have been awake if living on a 24-hr day for the same duration with 16 hours of wakefulness and 8 hours of sleep at their habitual time per day. We used this approach, rather than computing the actual amount of sleep obtained (which may be modulated by circadian phase), to follow the methods used in existing mathematical models of human performance, which base predictions on time in bed (i.e., sleep opportunity) [33,34]. The rationale for this approach is that such models could be applicable to real-world contexts where sleep opportunities are known but actual sleep duration cannot be reliably measured. This also is similar to a metric used by Van Dongen et al. [18] called Cumulative Excess Wakefulness which was calculated as the number of hours of wakefulness beyond a critical wake duration, statistically estimated to be 15.84 ± 0.73 hours [18].

Our goal in this study was to predict objective PVT, ADD, and DSST performance using VAS (*Alertness*) and three other variables: Circadian phase (*Phase*), hours since scheduled awakening (*Wake Duration*), and cumulative lost hours of sleep (*Sleep Debt*). Previous studies have shown that numerous physiological variables, including sleepiness, subjective alertness, and performance, depend on nonlinear interactions of circadian phase and sleep homeostatic pressure [11,17,19,24,30,35]. To determine the relationship between *Alertness* and PVT, ADD, or DSST, it is therefore important to use models that allow nonlinear relationships between these variables, rather than methods like Principal Component Analysis, which assume linear relationships between all variables.

After considering a variety of mathematical forms, we identified three candidate mathematical models that performed best in fitting the data. All models were mixed-effect models, to allow for individual participant differences. We call these models “Linear”, “Exponential”, and “Logistic”, based on the functional form of the term that incorporates the effects of *Phase* and *Wake Duration*. The form of these models is:

**Linear:** *P* = *b*_1_ + (*b*_2_*W + b*_3_)*X*
**Exponential:**$P=\;b_{1\;}+\left(b_{2\;}W+\;b_{3\;}\right)e^{b_{4}X}$
**Logistic:**$P=\;b_{1}+\left(b_{2}W+\;b_{3}\right)\frac{e^{\left(b_{4}\;+\;b_{5}X\right)}}{1+e^{\left(b_{4}\;+\;b_{5}X\right)}}$

where:

$$X=A+b_{6}\text{cos}\left(C+b_{7}\right)+b_{8}T$$

*b*_1_ − *b*_8_ are parameters to be fit,
*A* = VAS (*Alertness*),
*W* = Cumulative sleep loss in hours (*Sleep Debt*),
*P* = *log*(*PVT mean*), ADD Correct, or DSST Correct
*C* = Circadian Phase (*Phase*),
*T* = Hours since scheduled awakening (*Wake Duration*).

These three forms of the model were each fitted to the data using the MatLab functions nlinfit, which fits a nonlinear regression model, or nlmefit, which fits a nonlinear mixed effects regression model. Their goodness-of-fit was compared using an adjusted R^2^ metric.

In addition, we wanted to determine the relative importance of each of the four variables–*Alertness*, *Phase*, *Wake Duration*, and *Sleep Debt*–to predicting objective performance. To achieve this, we examined the effects of individually excluding or including each factor from the model, for all 16 possible combinations of the four variables. Variables were excluded by giving them a coefficient of zero. The Akaike Information Criterion (AIC) was used to compare models; AIC scores favor more parsimonious models. Lower AIC values indicate a better fit when adjustment is made for the number of variables in the model.

**Fig 1:**
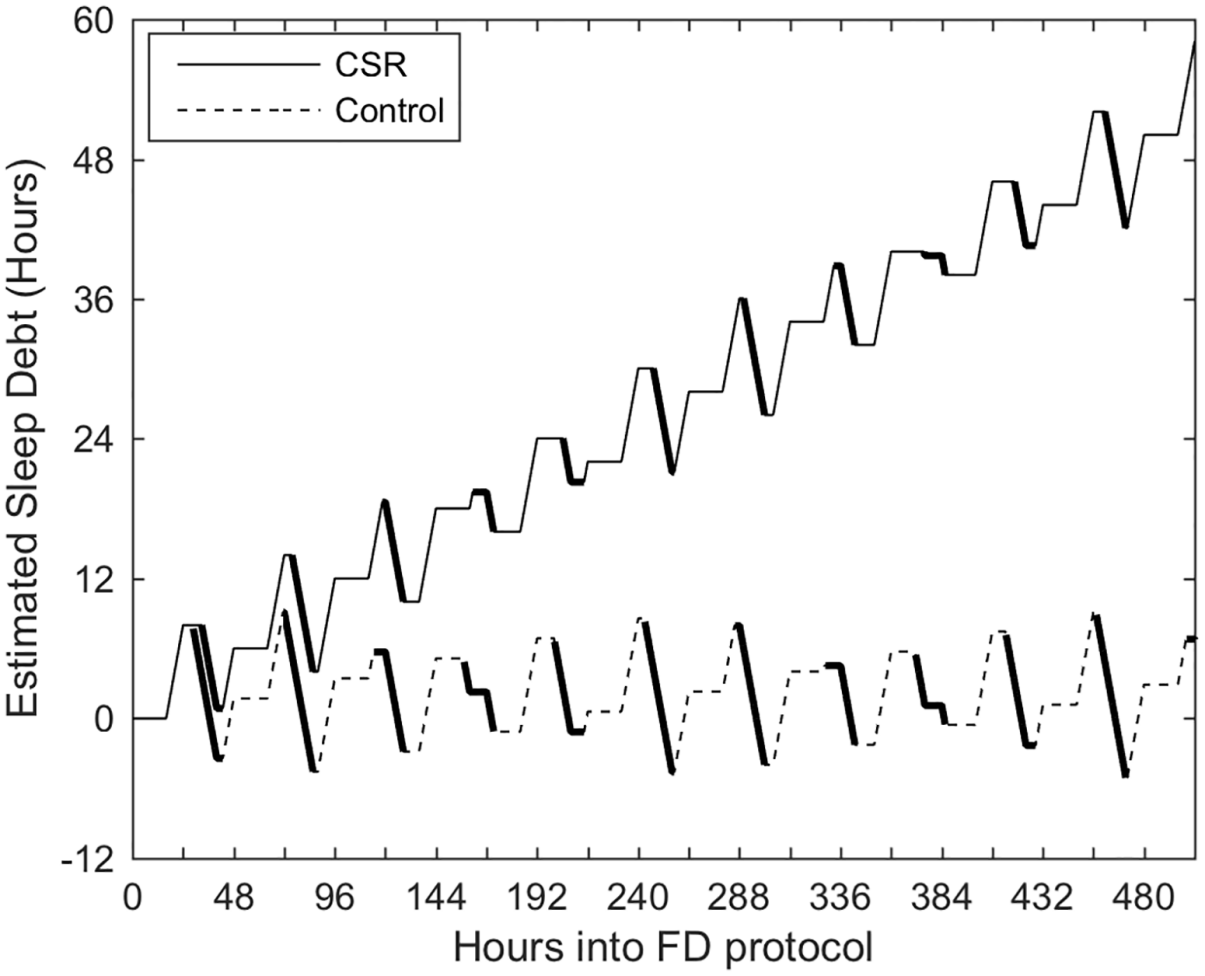
Changes in *Sleep Debt* by hour across the 3-week (twelve 42.85-h “days”) Forced Desynchrony protocol. *Sleep Debt* in the Control group varies around zero hours due to prolonged wake and sleep bouts with a 1:2 sleep:wake ratio. The Chronic Sleep Restriction (CSR) protocol accumulates *Sleep Debt* across the entire duration of the protocol. Heavy lines indicate when the sleep was scheduled.

*Sleep Debt* changed across the FD protocol for both Control and CSR groups, with over 50 hours of cumulative lost hours of sleep in the CSR group by the end of the FD protocol (Fig 1). We note that this variable is based on available sleep opportunities, and is therefore an estimate of the true amount of sleep debt.

Both PVT lapses (reaction time > 500 msec) and PVT mean reaction time for each test battery were investigated as metrics for PVT objective performance. A logarithmic transformation was applied to *PVT mean* reaction time and to the standard deviation of PVT reaction time *(PVT standard deviation)*. This was done to normalize the data (since they were not normally distributed) and make it easier to visualize the relationship between these variables, since the raw PVT metrics vary by orders of magnitude between well-rested and CSR conditions. This normalization achieves a similar outcome to taking reciprocal values of PVT reaction times, which has been used elsewhere [35]. The relationship between *log(PVT mean)* and *log*(*PVT standard deviation)* was modeled with a third degree polynomial using the Levenberg-Marquardt least squares algorithm and with mixed model regression.

For ADD and DSST test batteries the number of correct responses were used as metrics for objective performance.

## Results

**Fig 2:**
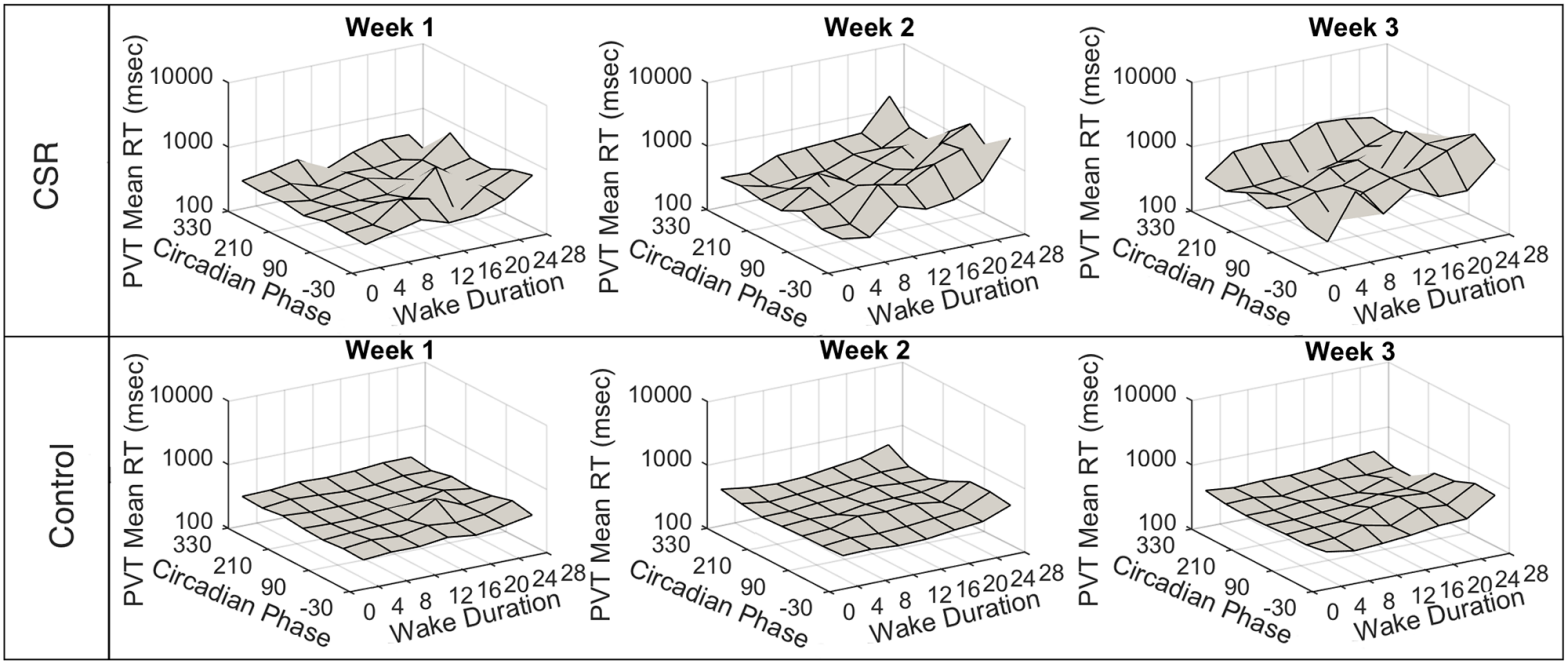
*PVT* scores by Circadian *Phase* and *Wake Duration*. Mean reaction times (RT) on the Psychomotor Vigilance Task (PVT) are plotted as a function of Circadian *Phase* (zero degrees is the fit melatonin maximum) and *Wake Duration* (hours elapsed in scheduled wake episode). The top panel contains data from the Chronic Sleep Restriction (CSR) condition. The bottom panel contains data from the Control condition. Each column shows one week (four 42.85-h “days”) of data.

**Fig 3:**
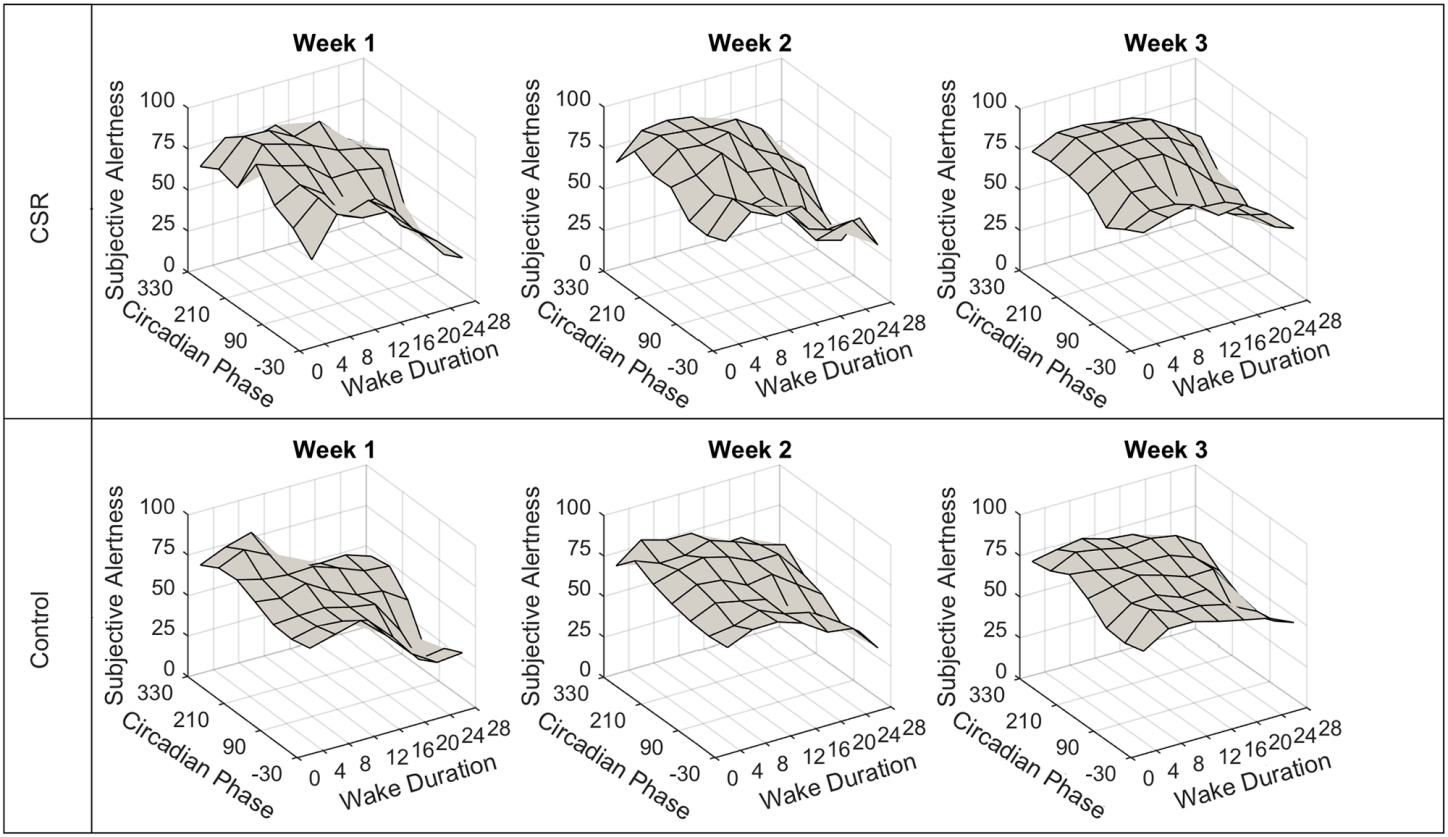
Subjective *Alertness* scores by Circadian *Phase* and *Wake Duration*. Mean Subjective *Alertness* scores are plotted as a function of Circadian *Phase* (zero degrees is the fit melatonin maximum) and *Wake Duration* (hours elapsed in scheduled wake episode). The top panel contains data from the Chronic Sleep Restriction (CSR) condition. The bottom panel contains data from the Control condition. Each column shows one week (four 42.85-h “days”) of data.

*PVT mean* and *PVT standard deviation* increased from week one to week three (reflecting a decrease in vigilant performance) in both Control and CSR. *PVT mean* values were 322±103ms in Control and 369±248ms in CSR in the first week, and were 593±796ms in Control and 1511±2106ms in CSR in the last week. *Alertness* ratings decreased in both groups between the first and third week: they were 65±17 in Control and 81±16 in CSR in the first week, and were 63±17 in Control and 53±27 in CSR in the third week. As reported by Cohen et al. [26] and shown in Fig 2, the slowest reaction times were observed at Adverse Time and Adverse Phase, with *mean PVT* reaction times of 571±584ms in Control and 2614±2962ms in CSR. The fastest reaction times were observed at Optimal Time and Optimal Phase, with *mean PVT* reaction times of 0306±61ms in Control and 297±154ms in CSR. Similarly, the lowest *Alertness* ratings were observed at Adverse Time and Adverse Phase, with mean rating of 42±23 in Control and 29±27 in CSR (Fig 3). The highest *Alertness* ratings were observed at Optimal Time and Optimal Phase, with 74±14 in Control and 83 ± 12 in CSR.

**Fig 4:**
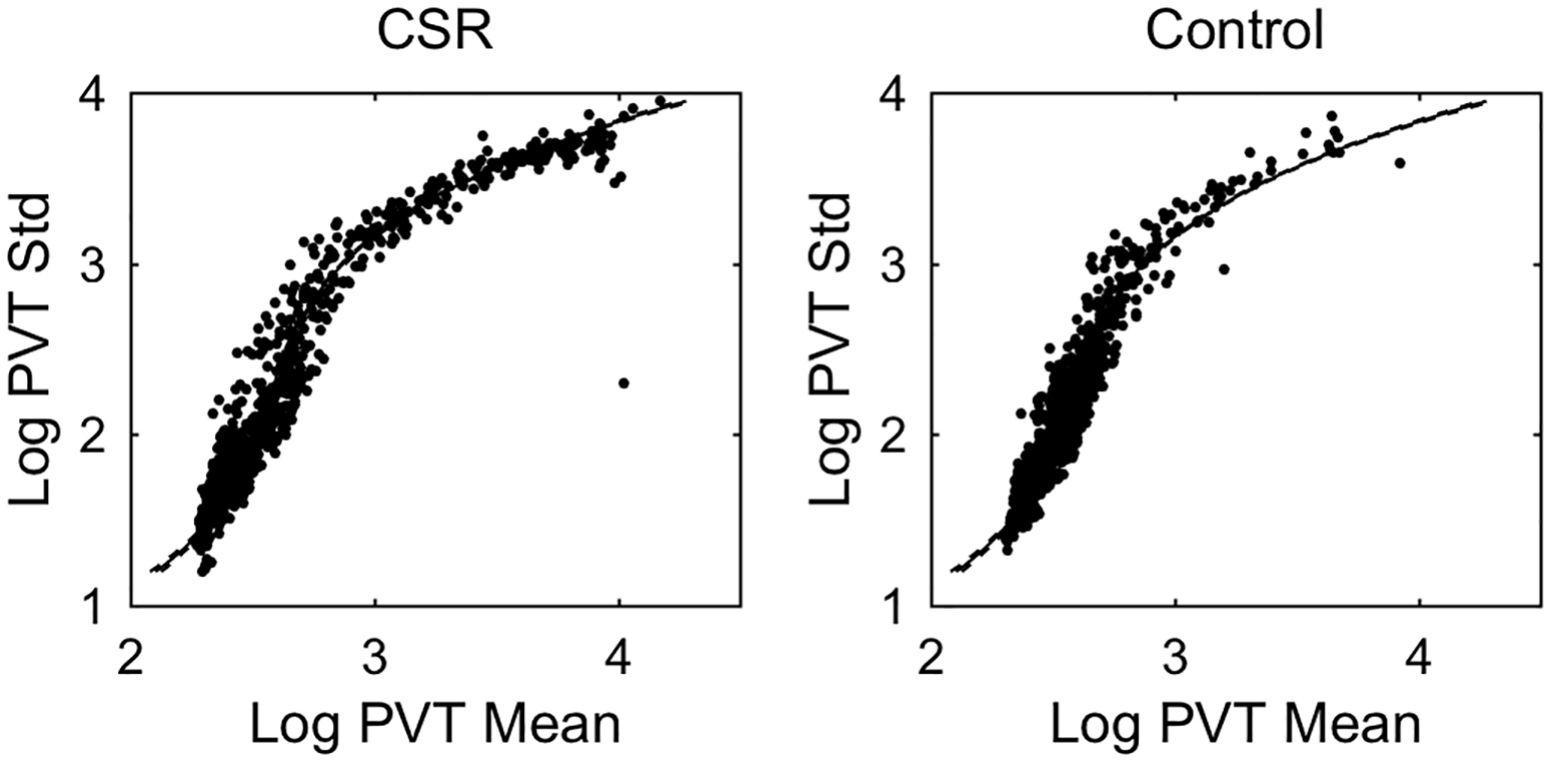
The relationship between *PVT standard deviation* and *PVT mean*. Data are shown for Chronic Sleep Restriction (CSR) and Control conditions. Data from both conditions demonstrate a robust relationship between the standard deviation and mean of the Psychomotor Vigilance Task (PVT). Logarithms of each variables are plotted to the wide ranges that the data span. Each data point represents one 10-min session. The same polynomial fit is shown to data in both plots with 95% confidence intervals indicated by dashed lines.

**Fig 5:**
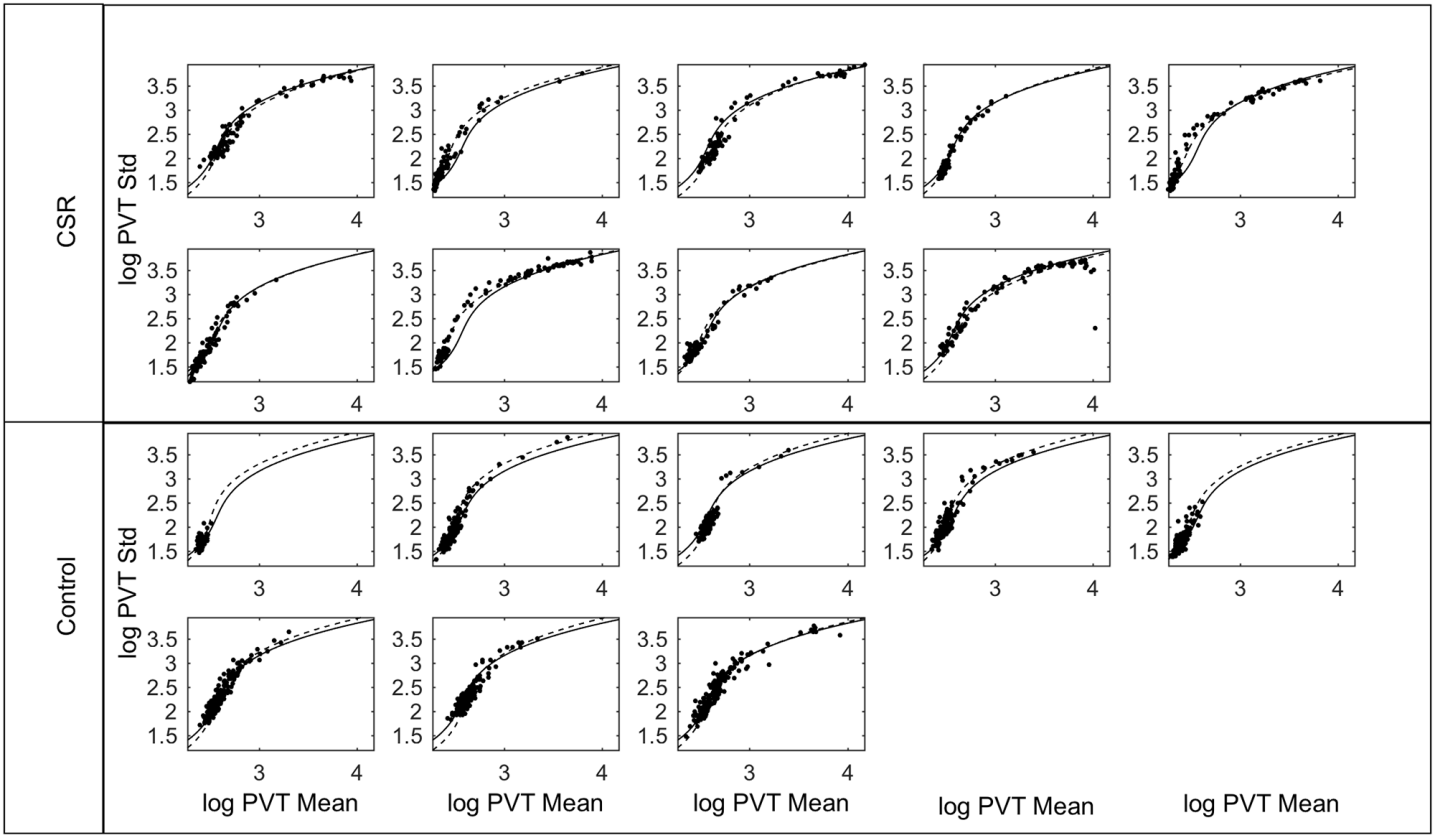
*PVT standard deviation* versus *PVT mean* for each participant. The same data as in Fig 4 are plotted separately for each participant. Polynomial fits are shown with fixed effects (solid black line) and with mixed effects (dashed line).

Across all test batteries, there was a positive relationship between *log(PVT mean)* and *log* (*PVT standard deviation)*. These variables were strongly correlated; a third degree polynomial fit yielded an adjusted R^2^ value of 0.947 (Fig 4). Notably, the relationship between these variables did not depend on experimental condition: the same curve describes the relationship in both Control and CSR conditions, with individuals in the CSR condition reaching higher points on the curve more often. There was little inter-individual variability in the relationship between these variables (Fig 5). Fitting the third order polynomial on an individual basis only marginally improved the adjusted R^2^ value to 0.964. Therefore, regardless of condition, if *log(PVT mean)* is known, accurate and reliable prediction of *log* (*PVT standard deviation)* is possible.

The frequency of lapses was also related to the participants' level of *Alertness* (Table 1). For *Alertness* in the lowest quintile (≤42.6), 92% of test batteries contained at least one PVT lapse; for *Alertness* in the highest quintile (≥83.4), only 56% of test batteries had at least one lapse. The crossover from more lapses than expected to fewer than expected occurs at ~70th percentile with values of *Alertness* of ~78.5. This contingency table has a χ^2^ of 305.4. The relative risk of having a lapse in a test battery between the lower and upper *Alertness* quintiles was 1.64 with a 95% confidence interval of 1.56–1.72 and p-value <0.001.

**Table 1:** Contingency Table of Alertness vs. Absence or Presence of PVT lapses.

Alertness	No Lapses	One or More Lapses	Row Totals
0–10% (0–22.8)	10 (42.9)	147 (114.1)	157
10–20% (22.8–42.6)	21 (58.8)	194 (156.2)	215
20–30% (42.6–53.3)	68 (99.9)	297 (265.1)	365
30–40% (53.3–62.8)	81 (111.1)	325 (294.9)	406
40–50% (62.8–68.5)	122 (164.7)	480 (437.3)	602
50–60% (68.5–73.9)	166 (187.9)	521 (499.1)	687
60–70% (73.9–78.5)	204 (204.6)	544 (543.4)	748
70–80% (78.5–83.4)	247 (223.2)	569 (592.8)	816
80–90% (83.4–100)	260 (187.4)	425 (497.6)	685
90–100% (89.4–100)	196 (94)	149 (250.6)	345
**Column Totals**	1375	3651	5026

Left column has *Alertness* rating by percentiles, with corresponding *Alertness* rating range in parentheses. In the **No Lapses** or **One or More Lapses** columns, number of occurrences is given and expected frequency (based on column total and row total) is given in parentheses.

**Fig 6:**
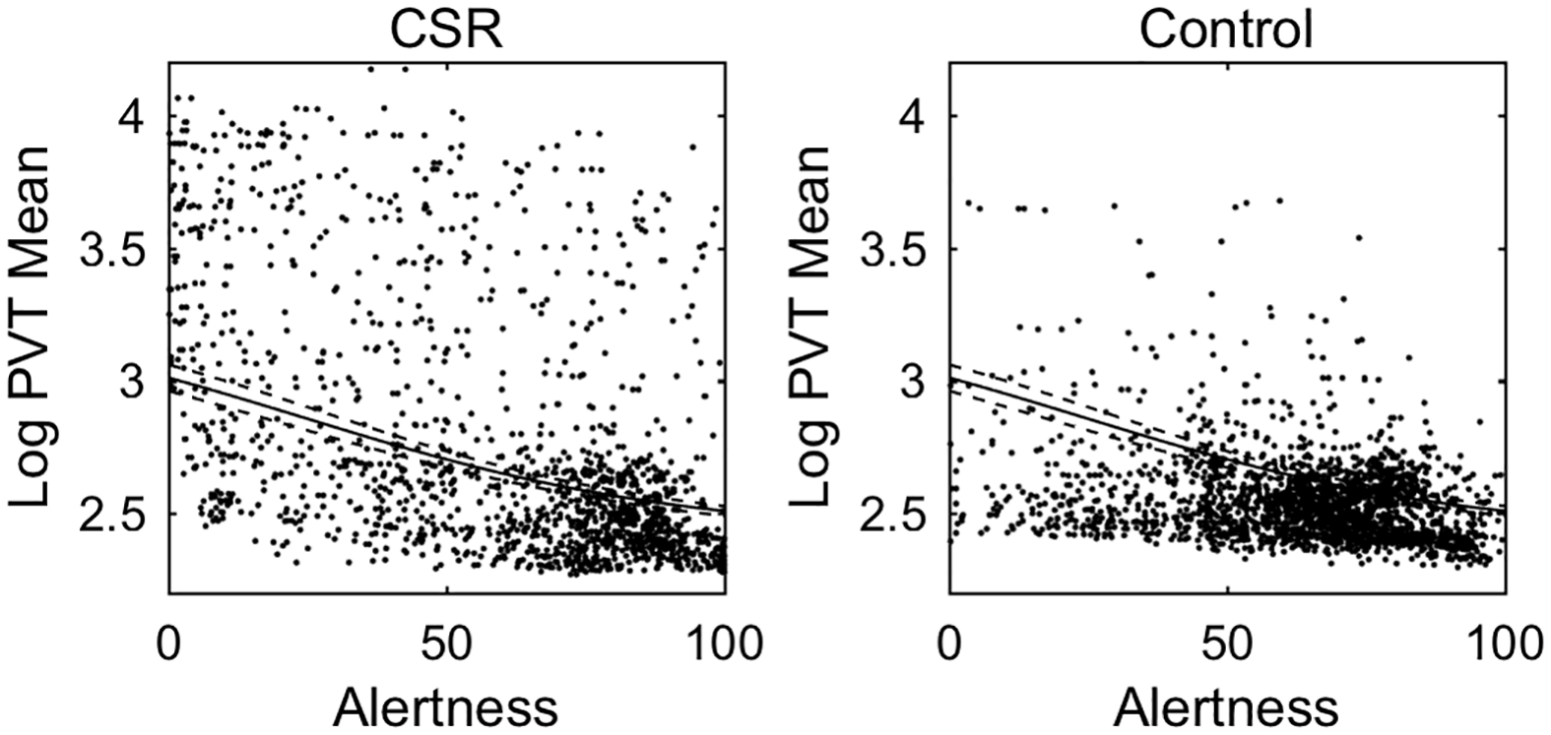
*PVT mean* vs. Subjective *Alertness*. Data are shown for Chronic Sleep Restriction (CSR) and Control conditions. Individual data points represent Subjective *Alertness* and mean Psychomotor Vigilance Task (PVT) responses from the same individual, paired by close timing in the same testing session. A single logistic function is fit to all data (solid line), with 95% confidence intervals of the best-fit line indicated by dashed lines.

**Fig 7:**
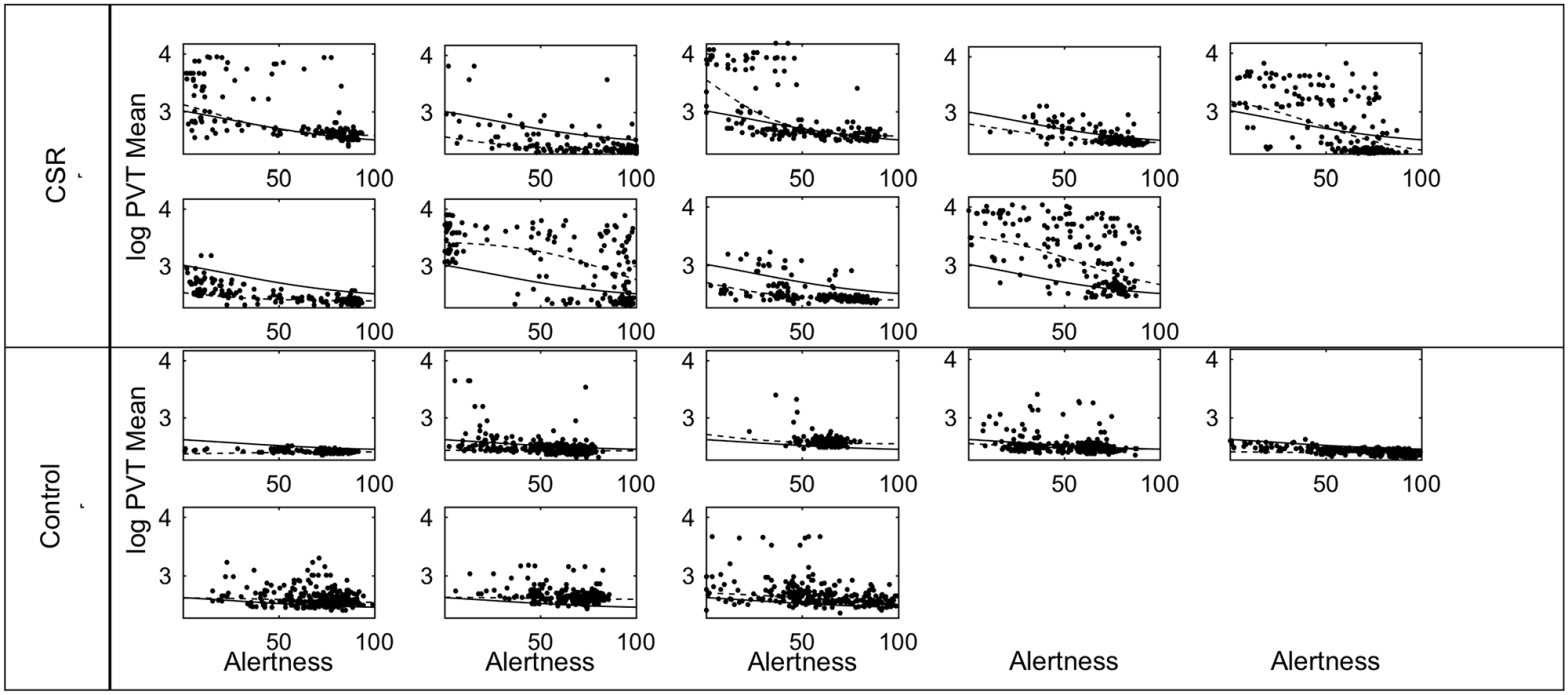
*PVT mean* vs. Subjective *Alertness* for each participant. The same data as in Fig 6 are plotted separately for each participant. Logistic fits are shown with fixed effects (solid black line) and with mixed effects (dashed line).

The relationship between *Alertness* and *PVT mean* reaction time is shown for all participants in Fig 6 and for each individual in Fig 7. With only *Alertness* in the model, there is an adjusted R^2^ of 0.557. Each *Alertness* rating had a wide range of associated PVT values. Overall, PVT reaction times were negatively correlated with *Alertness*. In the highest *Alertness* quintile, mean reaction time was 304±98ms in Control and 447±686ms in CSR. In the lowest *Alertness* quintile, mean reaction time was 574±725ms in Control and 2592±2898ms in CSR. Fitting linear, exponential, and logistic models to the group data resulted in adjusted R^2^ values of 0.338, 0.392, and 0.401, respectively. Some of the unexplained variability in these models is due to inter-individual differences, which is of key interest. Fitting these same models with mixed effects resulted in adjusted R^2^ values of 0.681, 0.703, and 0.728, respectively. Therefore, the logistic fit had the highest R^2^ values with and without subject-specific effects, although all three models performed similarly with subject-specific effects.

The AIC was used to compare different combinations of variables within the logistic model for PVT mean (Table 2). When models of only a single variable were considered, *Alertness* provided the lowest AIC value of 586. When two variables were considered, *Alertness* and *Sleep Debt* resulted in the lowest AIC value. This two-variable model has similar AIC values to the 3-variable models. When three variables were considered, *Alertness*, *Sleep Debt*, and *Wake Duration* had the lowest AIC. The lowest AIC value overall was obtained by including all four variables, meaning that each variable contains meaningful independent information.

**Table 2:** Table of Akaike Information Criterion (AIC) and Adjusted R^2^ values for the different predictive models of PVT mean.

Variables Used in the Predictive Model for PVT mean	Akaike Information Criterion (AIC) value	Adjusted R^2^
Phase	∝	0.352
Wake Duration	985	0.440
Sleep Debt	899	0.505
Alertness	586	0.529
Phase, Sleep Debt	919	0.4684
Phase, Alertness	573	0.482
Wake Duration, Sleep Debt	480	0.569
Wake Duration, Alertness	417	0.582
Wake Duration, Phase	393	0.606
Alertness, Sleep Debt	196	0.650
Phase, Alertness, Wake Duration	338	0.627
Phase, Alertness, Sleep Debt	195	0.663
Phase, Wake Duration, Sleep Debt	194	0.663
Wake Duration, Sleep Debt, Alertness	107	0.682
Phase, Wake Duration, Sleep Debt, Alertness	79	0.728

**Fig 8:**
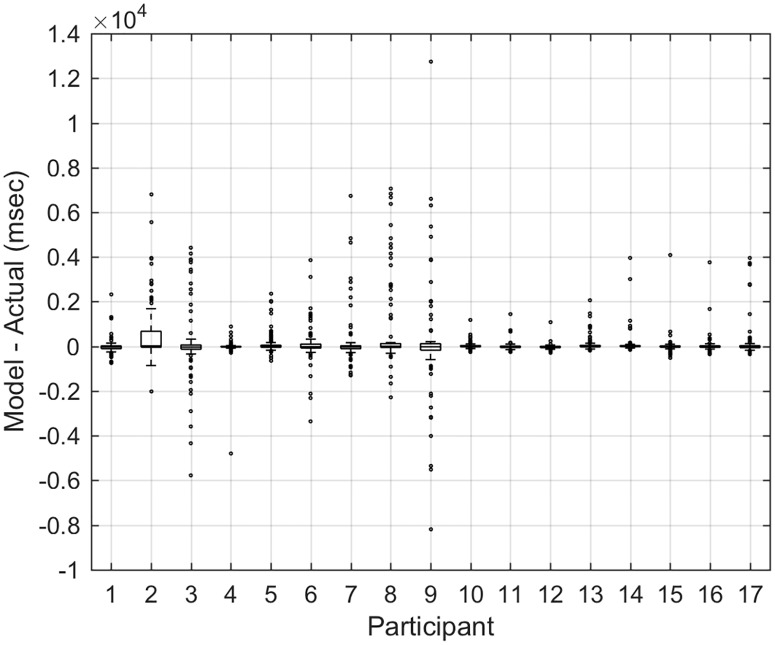
Comparison of model predictions of *PVT mean* relative to actual performance in each individual. For each participant, we show a box-plot of the differences between predicted Psychomotor Vigilance Task (PVT) mean in each 10-min session and actual PVT mean. Outliers are shown as dots. The model that includes all four variables is used for predictions. Participants 1–9 are from the Chronic Sleep Restriction (CSR) condition. Participants 10–17 are from the Control condition.

The model with only *Alertness* was not accurate at predicting PVT performance; it had a maximum underestimation of 13,467ms and maximum overestimation of 1097ms. The model with all variables provided the most accurate prediction of PVT performance, with a maximum underestimation of 1128ms and a maximum overestimation of 13ms. There was also a great deal of variability in the underestimation and overestimation for each participant (Fig 8): the standard deviations for participant underestimation and overestimation were 4576ms and 2412ms respectively. CSR participants had larger errors in estimation than Control participants; this underlines the significant contribution of the *Sleep Debt* parameter.

**Fig 9:**
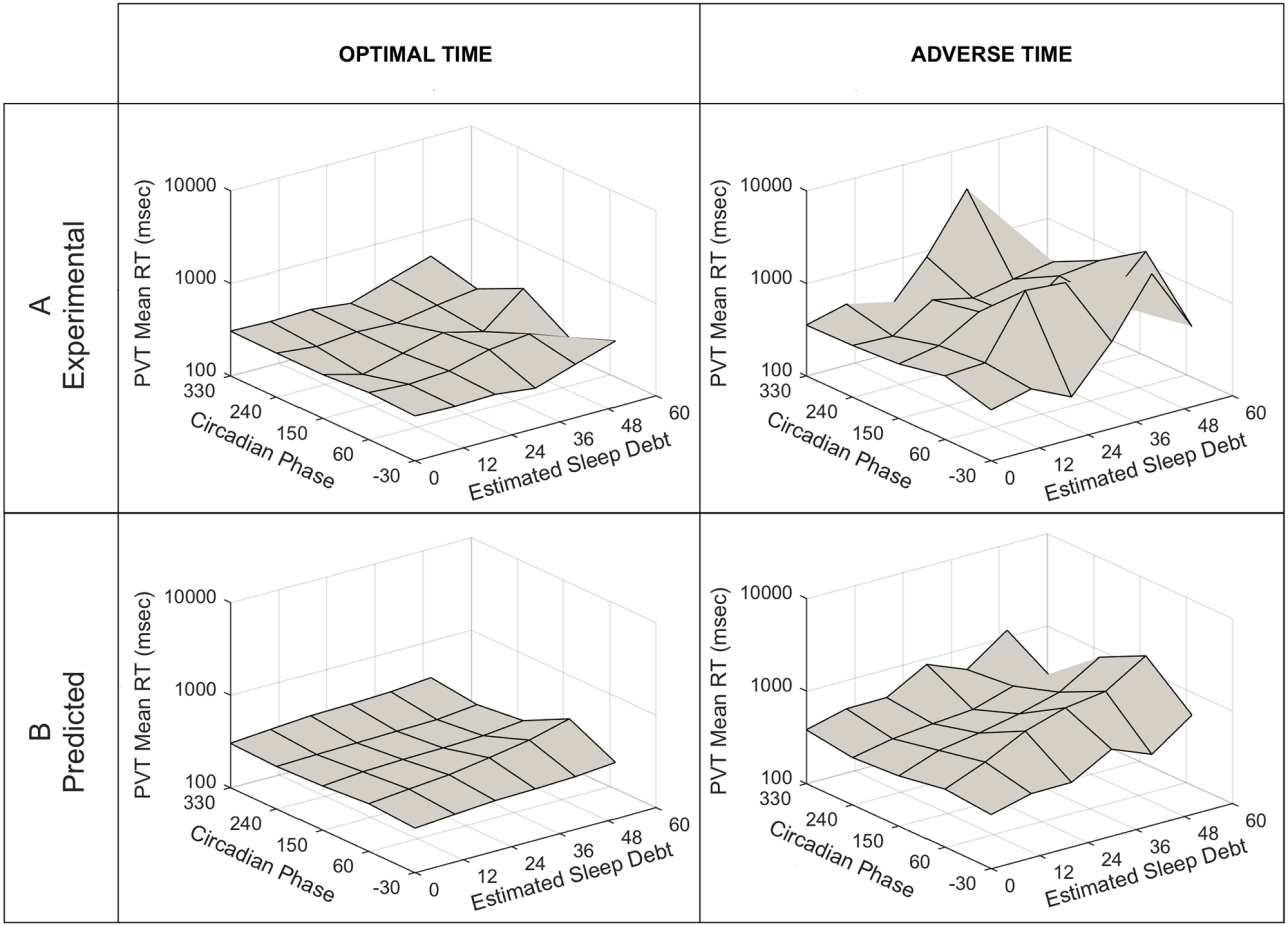
Top Row (A) Actual *PVT mean* for the experimental data vs. *Phase* and *Sleep Debt*. Bottom Row (B) Predicted *PVT mean* from model vs. *Phase* and *Sleep Debt*. Plots show the mean reaction time (RT) on the Psychomotor Vigilance Task (PVT) as a function of Circadian *Phase* (zero degrees is the fit melatonin maximum) and *Sleep Debt* (cumulative hours of insufficient sleep opportunity). Left column values are taken during “Optimal Time” (defined as hours 2–10 after awakening). Right column values are taken during “Adverse Time” (defined as hours 20–28 after awakening).

Using the logistic model with all variables, we tested the ability of *Alertness*, *Wake Duration*, *Phase*, *and Sleep Debt* to predict *PVT mean* reaction time (Fig 9). These predictions tended to underestimate the *log(PVT mean)* during Adverse Phases at Optimal Time and at the 50 hours of *Sleep Debt* bin center (Fig 9, left plot). Predictions were more accurate under conditions of Adverse Time and Adverse Phase and at 50 hours of *Sleep Debt*.

AIC values were obtained for ADD and DSST using the same three models, with values given in Tables 3 and 4, respectively. All AIC values were larger than those for the corresponding PVT models due to more data points for ADD and DSST, and thus a lower model likelihood. For ADD, when using a single variable, *Alertness* had the highest predictive value. However, the lowest AIC value for ADD was obtained with two variables, *Alertness* and *Phase*. *Wake Duration* and *Sleep Debt* did not improve AIC values for ADD. The variable combinations with the lowest AIC values were similar for DSST and PVT mean, across one, two, three, and four variables. For DSST, the model with all variables provided the lowest AIC value.

**Table 3:** Table of Akaike Information Criterion Values (AIC) for different predictive models of ADD.

Variables Used in the Predictive Model for ADD	Akaike Information Criterion (AIC) value
Slept Debt	29301
Wake Duration	29182
Phase	29149
Alertness	28977
Phase, Sleep Debt	29305
Wake Duration, Sleep Debt	29177
Wake Duration, Phase	29049
Alertness, Sleep Debt	28979
Wake Duration, Alertness	28965
Phase, Alertness	28921
Wake Duration, Sleep Debt, Alertness	28966
Phase, Wake Duration, Sleep Debt	28963
Phase, Alertness, Wake Duration	28922
Phase, Alertness, Sleep Debt	28922
Phase, Wake Duration, Sleep Debt, Alertness	28924

**Table 4:** Table of Akaike Information Criterion Values (AIC) for different predictive models of DSST.

Variables Used in the Predictive Model for DSST Correct	Akaike Information Criterion (AIC) value
Sleep Debt	26230
Phase	26123
Wake Duration	26094
Alertness	26025
Phase, Sleep Debt	26234
Wake Duration, Alertness	26024
Wake Duration, Sleep Debt	26022
Phase, Alertness	26006
Wake Duration, Phase	25994
Alertness, Sleep Debt	25921
Phase, Alertness, Wake Duration	25972
Phase, Wake Duration, Sleep Debt	25936
Phase, Alertness, Sleep Debt	25929
Wake Duration, Sleep Debt, Alertness	25918
Phase, Wake Duration, Sleep Debt, Alertness	25900

## Discussion

### The relation between PVT performance and subjective alertness

This study confirms that *Alertness* does correlate with PVT performance within a testing session, and it is the best individual predictor of PVT, ADD, and DSST. This is an interesting result considering that unlike *Phase*, *Wake Duration*, or *Sleep Debt*; *Alertness* is the only predictor that is not objectively determined. However, *Alertness* alone is an inaccurate predictor for an individual.

Others studies have found a similar relationship in group averages between subjective ratings of sleepiness and objective measures of neurobehavioral performance. Kaida et al. [36] found that the Karolinska Sleepiness scale was correlated with PVT lapses (r = 0.56) and strongly correlatedwith VAS *Alertness* (r = 0.89). Dorrian et al. [37] suggest that VAS *Alertness* may be a global subjective assessment of cognitive performance, because this single rating of subjective alertness given before a group of tests was correlated with all pretest predictions by participants on 6 measures of cognitive performance. Pretest predictions were also correlated with actual performance on 3 of the 6 measures.

The tendency for subjective alertness to underestimate vigilant performance has also been noted in other studies [24]. In our study, subjects’ subjective sense of impairment deviated most from PVT performance during the Adverse Phase (i.e., biological night). Similar results were noted by Zhou et al. [24] who used standardized PVT and VAS scores and found a greater deviation between PVT and VAS during biological night. We extend this finding, noting that subjective *Alertness* was particularly inaccurate at predicting vigilance in the setting of adverse circadian phase and chronic sleep debt, even when wake duration was only 2–10 hours. This scenario would be particularly common in night-shift workers, who may therefore have a false sense of reassurance about their own ability to remain vigilant when they have recently slept. Note that participants were least able to accurately rate their vigilant performance during times when they are most likely to suffer from impairment.

Leproult et al. [38] found that, while the temporal profiles of decrements in individual participant’s subjective alertness and objective performance are similar (since they are under the dual control of circadian and homeostatic processes), the magnitudes of their impairments are unrelated. Several of their participants demonstrated better cognitive performance than their subjective fatigue rating would suggest. This result may have been due to using a shorter performance task duration of only 4 minutes in their study, however. Variability in subjective ratings of performance is also due to individual differences in tolerance to sleep restriction [39,40]. Our predictions improved remarkably when mixed effect models were used. Models of PVT without mixed effects had an adjusted R^2^ of 0.401 while the R^2^ was 0.728 with mixed effects.

The best prediction of PVT and DSST performance took into account subjective alertness, circadian phase, time since awakening, cumulative amount of sleep loss, and individual effects. In terms of practical value, *Alertness* and *Wake Duration* are relatively easy measures to obtain. Gathering data for *Sleep Debt* may require accurate logs or participant estimates. This extra effort may be worthwhile since *Sleep Debt* was the second best sole predictor of PVT performance. Determining *Phase* is most difficult in the field, but could be estimated based on prior sleep history. This variable was not a strong predictor for PVT or ADD performance, but was the second best predictor for DSST performance. The importance of obtaining an accurate measure of *Phase* will therefore depend on the nature of the operational demands.

### Limitations

It is possible that participants are unable to accurately predict PVT because of the short duration of time required to record the VAS, (i.e., a few seconds), which may cause participants to rate their ability in relation to a short duration task, rather than taking into account the vigilance required for a 10 minute PVT test. It has been shown that time on a task does affect attention and performance [41]. Some authors [36,42] have suggested asking participants to sit quietly for one minute before filling out the VAS in order to give them some time to reflect on their answer. Another approach may be to utilize a scale that asks participants to rate their ability to remain alert during a 10-minute task; it would be useful to determine if using this approach would improve the accuracy of subjective sleep ratings.

An additional possible confounding factor is that participants in the Control group underwent the PVT test more frequently than the CSR group. Since the PVT is a 10-minute test, more frequent testing may lead to monotony and a decrease in motivation in the Control group. However, this would be expected to underestimate the effect of CSR, which is not what was seen.

Finally, we used sleep opportunities during FD as the basis for calculating *Sleep Debt* and *Wake Duration*, rather than the actual sleep periods obtained. This is important, because individuals may not have slept for the entire sleep opportunity, especially when sleep opportunities occurred during the biological day. We used this approach because we wanted the models to be applicable to real-world data and it is easier in the real world to obtain estimates of time spent in bed rather than estimates of the sleep duration. In addition, actual sleep efficiency [(total sleep time/time in bed) x 100%] of 84% in the Control group and 90% in the CSR group during FD are comparable to typical sleep efficiencies expected in the field, supporting the generalizability of these findings.

### Future steps and models used in the workplace

Further study is needed to determine whether a similar model to the one presented in this paper could be generalized to predict performance on more complicated cognitive tasks. The implementation of individual effects improves predictions, but complicates the utility of the model in practice. One future step could include integration of mobile or wearable devices with software for analysis and fatigue-based risk management. Such collection systems could modify predictions using Bayesian forecasting procedures, as described by Van Dongen et al. [43]. Ultimately, the utility of the model will depend on the setting in which it is used [44,45]. Bio-mathematical models can give an estimate of the degree of performance impairment and the relative likelihood of making a mistake. The actual cost of that mistake depends upon the work environment and task.

## Conclusions

Given the negative association between chronic sleep restriction history and safety, it is important to find a metric that can predict conditions where performance is likely impaired. This is a pervasive problem, as up to 40% of adults are sleeping fewer than 7 hours on weeknights [46]. We have shown that while most individuals are partially aware of their impairment with CSR, they are not fully cognizant of its detrimental effects on their vigilant performance. Therefore, reliance on a VAS subjective *Alertness* rating is insufficient to accurately predict cognitive performance as measured by PVT, ADD or DSST. In addition, a predictive model should include individual variation, as the ability to predict objective performance dramatically improves when mixed effects models are involved. This may be due to the fact that subjective *Alertness* is highly specific to the individual and also that people vary in their sensitivity to *Sleep Debt*. The best predictive model of cognitive performance using PVT and DSST as a metric was obtained when *Alertness*, *Phase*, *Wake Duration*, and *Sleep Debt* were included with mixed effects.

While most participants are not fully aware of deficits in their cognitive performance, it may be that education regarding these factors would improve ability to predict cognitive performance. It has been noted that a subject’s rating accuracy improves when given tasks that provide feedback on cognitive performance [23]. In the future, better comprehension of the biological processes involved in sleep restriction may help to refine predictive models of cognitive performance. In addition, consideration of these factors when designing duty-hour regulations and work schedules and public education of the inadequacy of self-assessed alertness relative to performance, could improve occupational health and safety.

## References

[1] Ulmer C, Miller Wolman D, Johns MME. Resident Duty Hours: Enhancing Sleep, Supervision, and Safety, 1st ed. Washington (DC): National Academies Press; 2009. 25009922

[2] Gander P, Millar M, Webster C, Merry A. Sleep loss and performance of anaesthesia trainees and specialists. Chronobiol Int. 2008;25: 107–91. 10.1080/07420520802551428 19005906

[3] Caldwell JA Jr. Fatigue in the aviation environment: an overview of the causes and effects as well as recommended countermeasures. Aviat Space Environ Med. 1997;68: 932–8. 9327120

[4] Dalziel JR, Soames-Job RF. Risk-taking and fatigue in taxi drivers In Harley I., editor. Managing Fatigue in Transportation. Oxford Elsevier Science Ltd 1998; pp. 287–97.

[5] Costa G. The impact of shift and night work on health. Appl Ergonom. 1996;27: 9–16. 10.1016/0003-6870(95)00047-x 15676307

[6] Akerstedt T. Shift work and disturbed sleep/wakefulness. Sleep Med Rev. 1998;2:117–28. 15310506 10.1016/s1087-0792(98)90004-1

[7] Komada Y, Asaoka S, Abe T, Inoue Y. Short sleep duration, sleep disorders, and traffic accidents. IATSS Research. 2013;37: 1–7.

[8] Philip P, Sagaspe P, Moore N, Taillard J, Charles A, Guilleminault C, et al Fatigue, sleep restriction and driving performance. Accid Anal Prev. 2005;37: 473–8. 15784201 10.1016/j.aap.2004.07.007

[9] Belenky G, Wesensten NJ, Thorne DR, Thomas ML, Sing HC, Redmond DP, et al Patterns of performance degradation and restoration during sleep restriction and subsequent recovery: a sleep dose—response study. J Sleep Res. 2003;12: 1–12. 12603781 10.1046/j.1365-2869.2003.00337.x

[10] Neylan TC, Metzler TJ, Henn-Haase C, Blank Y, Tarasovsky G, McCaslin SE, et al Prior night sleep duration is associated with psychomotor vigilance in a healthy sample of police academy recruits. Chronobiol Int. 2010;27: 1493–508. 10.3109/07420528.2010.504992 20795888 PMC13003608

[11] Van Dongen HPA, Dinges DF. Sleep, Circadian Rhythms and Psychomotor Vigilance. Clinics in Sports Medicine. 2005;24: 237–49. 15892921 10.1016/j.csm.2004.12.007

[12] Silva EJ, Wang W, Ronda JM, Wyatt JK, Duffy JF. Circadian and wake-dependent influences on subjective sleepiness, cognitive throughput, and reaction time performance in older and young adults. Sleep. 2010:33: 481–90. 20394317 10.1093/sleep/33.4.481 PMC2849787

[13] Wright KP, Hull JT, Czeisler CA. Relationship between alertness, performance, and body temperature in humans. Am J Physiol, 2002;283: R1370–R1377. 10.1152/ajpregu.00205.2002 12388468

[14] Pilcher JJ, Huffcutt AI. Effects of sleep deprivation on performance: a meta-analysis. Sleep 1996;19: 318–26. 8776790 10.1093/sleep/19.4.318

[15] Goel N, Rao H, Durmer J, Dinges DF. Neurocognitive Consequences of Sleep Deprivation. Semin Neurol. 2009;29: 320–39. 10.1055/s-0029-1237117 19742409 PMC3564638

[16] Thomas M, Sing H, Belenky G, Holcomb H, Mayberg H, Dannais R, et al Neural basis of alertness and cognitive performance impairments during sleepiness. J Sleep Res. 2000;9: 335–52. 11123521 10.1046/j.1365-2869.2000.00225.x

[17] Pomplun M, Silva E, Ronda JM, Cain SW, Munch MY, Czeisler CA, et al The effects of circadian phase, time awake, and imposed sleep restriction on performing complex visual tasks: Evidence from comparative visual search. J Vision. 2012;12: 14. 10.1167/12.7.14 PMC4503214 22836655

[18] Van Dongen HPA, Maislin G, Mullington JM, Dinges DF. The cumulative cost of additional wakefulness: dose-response effects on neurobehavioral functions and sleep physiology from chronic sleep restriction and total sleep deprivation. Sleep. 2003;26: 117–26. 12683469 10.1093/sleep/26.2.117

[19] Wyatt JK, Ritz-De Cecco A, Czeisler CA, Dijk DJ. Circadian temperature and melatonin rhythms, sleep, and neurobehavioral function in humans living on a 20-h day. Am J Physiol 1999;277: R1152–63. 10516257 10.1152/ajpregu.1999.277.4.r1152

[20] Harrison Y, Home JA. The impact of sleep on decision making: a review. J Exp Psychol Appl. 2000;6: 236–49. 11014055 10.1037//1076-898x.6.3.236

[21] Brown ID. Driver fatigue. Hum Factors. 1994;36: 298–314. 8070794 10.1177/001872089403600210

[22] Dalziel JR, Soames-Job RF. Risk-taking and fatigue in taxi drivers In Harley I., editor. Managing Fatigue in Transportation. Oxford Elsevier Science Ltd 1998: pp 287–97.

[23] Dorrian J, Lamond N, Holmes AL, Burgess HJ, Roach GD, Fletcher A, et al The ability to self-monitor performance during a week of simulated night shifts. Sleep. 2003;26: 871–77. 14655922 10.1093/sleep/26.7.871

[24] Zhou X, Ferguson SA, Matthews R, Sargent C, Darwent D, Kennaway DJ, et al Mismatch between subjective alertness and objective performance under sleep restriction is greatest during the biological night. J Sleep Res. 2012;21: 40–9. 10.1111/j.1365-2869.2011.00924.x 21564364

[25] Wyatt JK, Cajochen C, Ritz-De Cecco A, Czeisler CA, Dijk DJ. Low-dose repeated caffeine administration for circadian-phase—dependent performance degradation during extended wakefulness. Sleep. 2004;27: 374–81. 15164887 10.1093/sleep/27.3.374

[26] Cohen DA, Wang W, Wyatt JK, Kronauer RE, Dijk DJ, Czeisler CA, et al Uncovering residual effects of chronic sleep loss on human performance. Sci Transl Med. 2010;2: 14ra3 10.1126/scitranslmed.3000458 20371466 PMC2892834

[27] Dinges DF, Powell JW. Microcomputer analyses of performance on a portable, simple visual RT task during sustained operations. Behav. Res. Meth. Instrum Comput. 1985;17: 652–55.

[28] Dorrian J, Rogers NL, Dinges DF. Psychomotor vigilance performance: neurocognitive assay sensitive to sleep loss In: Kushida CA, editor, Sleep Deprivation. Clinical Issues, Pharmacology, and Sleep Loss Effects. Marcel Dekker, New York, 2005: pp 39–70.

[29] Lim J, Dinges DF. A meta-analysis of the impact of short-term sleep deprivation on cognitive variables. Psychol Bull. 2010;1: 375–89. 10.1037/a0018883 PMC3290659 20438143

[30] Dijk DJ, Duffy JF, Czeisler CA. Circadian and sleep/wake dependent aspects of subjective alertness and cognitive performance. J Sleep Res. 1992;1: 112–7. 10607036 10.1111/j.1365-2869.1992.tb00021.x

[31] Czeisler CA, Duffy JF, Shanahan TL, Brown EN, Mitchell JF, Rimmer DW, et al Stability, precision, and near-24-hour period of the human circadian pacemaker. Science. 1999;284: 2177–81. 10381883 10.1126/science.284.5423.2177

[32] Czeisler CA, Buxton OM, Khalsa SBS. The human circadian timing system and sleep-wake regulation In: Kryger MH, Roth T, Dement WC, editors. Principles and Practices of Sleep Medicine. 5th ed Elsevier Saunders, St. Louis, MO; 2010 pp 375–394.

[33] McCauley P, Kalachev LV, Smith AD, Belenky G, Dinges DF, Van Dongen HPA. A new mathematical model for the homeostatic effects of sleep loss on neurobehavioral performance. J Theor Biol. 2009;256: 227–39. 10.1016/j.jtbi.2008.09.012 18938181 PMC2657297

[34] Rajdev P, Thorsley D, Rajaraman S Rupp TL, Wesensten NJ, Balkin TJ, et al A unified mathematical model to quantify performance impairment for both chronic sleep restriction and total sleep deprivation. J Theor Biol., 2013;331: 66–77. 10.1016/j.jtbi.2013.04.013 23623949

[35] Benitez PL, Kamimori GH, Balkin TJ, Greene A, Johnson ML. Modeling Fatigue over Sleep Deprivation, Circadian Rhythm, and Caffeine with a Minimal Performance Inhibitor Model. Methods Enzymol. 2009;454: 405–21. 10.1016/S0076-6879(08)03816-0 19216936 PMC2654588

[36] Kaida K, Takahashi M, Akerstedt T, Nakata A, Otsuka Y, Haratani T, et al Validation of the Karolinska sleepiness scale against performance and EEG variables. Clin Neurophysiol. 2006;117: 1574–1581. 16679057 10.1016/j.clinph.2006.03.011

[37] Dorrian J, Lamond N, Dawson D. The ability to self-monitor performance when fatigued. J Sleep Res. 2000;9: 137–44. 10849240 10.1046/j.1365-2869.2000.00195.x

[38] Leproult R, Colecchia EF, Berardi AM, Stickgold R, Kosslyn SM, Van Cauter E. Individual differences in subjective and objective alertness during sleep deprivation are stable and unrelated. Am J Physiol. 2003;284: 280–90. 10.1152/ajpregu.00197.2002 12529281

[39] Van Dongen HPA, Baynard MD, Maislin G, Dinges DF. Systematic interindividual differences in neurobehavioral impairment from sleep loss: evidence of trait-like differential vulnerability. Sleep. 2004;27: 423–33. 15164894

[40] Lo JC, Groeger JA, Santhi N, Arbon EL, Lazar AS, Hasan S, Von et al Effects of partial and acute total sleep deprivation on performance across cognitive domains, individuals and circadian phase. PLOS One 2012;7: e45987 10.1371/journal.pone.0045987 23029352 PMC3454374

[41] Otmani S, Pebayle T, Roge J, Muzet A. Effect of driving duration and partial sleep deprivation on subsequent alertness and performance of car drivers. Physiol Behav. 2005;84: 715–24. 15885247 10.1016/j.physbeh.2005.02.021

[42] Short M, Lack L, Wright H. Does Subjective Sleepiness Predict Objective Sleep Propensity? Sleep. 2010;33: 123–9 20120629 10.1093/sleep/33.1.123 PMC2802239

[43] Van Dongen HPA, Mott CG, Huang J, Mollicone DJ, McKenzie FD, Dinges DF. Optimization of Biomathematical Model predictions for Cognitive Performance Impairment in Individuals: Accounting for Unknown Traits and Uncertain States in Homeostatic and Circadian Processes. Sleep. 2007;30: 1129–43. 17910385 10.1093/sleep/30.9.1129 PMC1978411

[44] Dawson D, Noy I, Harma M, Akerstedt T, Belenky G. Modelling fatigue and the use of fatigue models in work settings. Accid Anal Prev. 2011;43: 549–64. 10.1016/j.aap.2009.12.030 21130216

[45] Jewett MD, Kronauer RE. Interactive mathematical models of subjective alertness and cognitive throughput in humans. J Biol Rhythms. 1999:14: 588–97. 10643756 10.1177/074873099129000920

[46] Centers for Disease Control and Prevention. Effect of short sleep duration on daily activities—_ United States, 2005–2008. MMWR Morb Mortal Wkly Rep. 2011;60: 239–42. 21368739

